# Complete genome sequence of *Bacillus paramobilis* sp. strain IMGN7 from soil

**DOI:** 10.1128/mra.00540-24

**Published:** 2024-07-31

**Authors:** Hoseong Jung, Sejin Choi, Yeongjun Kim, Jeong A. Han, Ho-Seok Lee, Eun Yu Kim

**Affiliations:** 1 Center for Genome Engineering, Institute for Basic Science, Daejeon, South Korea; 2 Department of Biology, College of Sciences, Kyung Hee University, Seoul, South Korea; 3 Gyeonggido Agricultural Research & Extension Services, Hwaseong, South Korea; 4 Division of Natural and Applied Sciences, Duke Kunshan University, Kunshan, Jiangsu, China; 5 Environment Research Center, Duke Kunshan University, Kunshan, Jiangsu, China; DOE Joint Genome Institute, Berkeley, California, USA

**Keywords:** *Bacillus*, *Bacillus paramobilis*, biocontrol agent, bioremediation

## Abstract

Here, we report the complete genome sequence of *Bacillus paramobilis* sp. strain IMGN7, which was isolated from soil in South Korea. Its complete genome size is 5.28 Mbp. This genome will provide various insights for further studies about their function as biocontrol agents, such as bioremediation and antibiosis.

## ANNOUNCEMENT

*Bacillus paramobilis* is facultatively anaerobic, rod-shaped, non-motile, and gram-positive (1). It has been isolated on the rhizosphere of cotton in Alabama, USA (2). Additionally, it has been predominantly isolated from soil contaminated with trinitrotoluene (TNT) (3). *B. paramobilis* has demonstrated a significant capacity to degrade not only TNT but also its byproducts (3). Given these attributes, our analysis of the strain isolated from soil provides additional insights into the functionality of similar strains, particularly in the realms of bioremediation and biocontrol.

The new strain, *B. paramobilis* sp. strain IMGN7, was isolated from soil collected in Danwolmyeon, Yangpyeong, Gyeonggi-do, South Korea, in 2019. It was isolated from a soil sample and spread on the tryptic soy agar plate. Incubation was performed at 15°C for 16 hours in dark conditions, and the sample was streaked twice to obtain a single colony.

Genomic DNA was extracted from a single colony using the Maxwell RSC Tissue DNA kit protocol. The extracted genomic DNA was divided into two parts for different sequencing methods. First, one part was sheared with the Megaruptor 3 (Diagenode) and purified using AMPure PB magnetic beads (Pacific Biosciences) for size selection. The PacBio sequencing library was prepared using the PacBio SMRTbelll prep kit 3.0. Subsequently, HiFi sequencing was performed on the PacBio Sequel II, generating 100,436 reads. The mean length of HiFi reads was 9,137 base pairs, with an N50 value of 9,740 base pairs.

Second, the other part was used to prepare the Illumina sequencing library using the TruSeq DNA Nano Library Prep Kit (Illumina, Inc., San Diego, CA, USA). Additional sequencing was conducted on the Illumina Hiseq X Ten platform with a 2 × 150 bp paired-end protocol, yielding 7,622,902 reads. Subsequently, quality filtering and adaptor trimming were performed using Trimmomatic v0.38 (4), ensuring a phred score of 30 or higher in 90% of the bases.

The HiFi reads were assembled *de novo* with HGAP v4 (5) to generate the complete genome assembly. Following the assembly, Illumina reads were used to polish the genome with Pilon v1.21 (6) three times. The final genome of *Bacillus paramobilis* IMGN7 consisted of a single contig, totaling 5,282,002 base pairs, with an average GC content of 35.6%, N50 value of 5,286,002, and coverage depth of 173.5. To assess assembly quality, BUSCO v5.1.3 (7) analysis was performed and found it highly complete (99.19%).

Genome annotation was performed using the NCBI Prokaryotic Genome Annotation Pipeline (v6.7) (8). We identified 5,414 predicted genes, including 5,261 coding sequences, 106 tRNAs, and 42 rRNAs. The genome annotation indicates various functional genes expected to perform biocontrol functions, including the production of chitinase, phenazine, HCN, and siderophore (9). In conclusion, this genome information will provide further insight into this species’ abilities in biocontrol and bioremediation.

## Data Availability

The annotated complete genome sequences of *B. paramobilis* sp. strain IMGN7 have been deposited at DDBJ/ENA/GenBank under the accession CP151108, and the version described in this paper is version CP151108.1. The raw reads are available under the BioProject accession number PRJNA1096151, and the BioSample accession number is SAMN40747984. The sequence data obtained in this work have been deposited in the NCBI Sequence Read Archive under the accession numbers SRX24247164 and SRX24247165.
